# Trans‑anal minimally invasive surgery (TAMIS) versus rigid platforms for local excision of early rectal cancer: a systematic review and meta-analysis of the literature

**DOI:** 10.1007/s00464-024-11065-6

**Published:** 2024-07-18

**Authors:** Zoe Garoufalia, Peter Rogers, Sarinya Meknarit, Sofoklis Mavrantonis, Pauline Aeschbacher, Emeka Ray-Offor, Sameh Hany Emile, Rachel Gefen, Justin Dourado, Nir Horesh, Steven D. Wexner

**Affiliations:** 1 Ellen Leifer Shulman and Steven Shulman Digestive Disease Center, Cleveland Clinic Florida, 2950 Cleveland Clinic Blvd, Weston, FL 33331 USA; 2 FAU Charles E. Schmidt College of Medicine, Boca Raton, FL USA; 3 Bart’s and the London School of Medicine and Dentistry, London, UK; 4 Department of General Surgery and Bariatric and Metabolic Institute, Cleveland Clinic Florida, Weston, FL USA; 5 Department for Visceral Surgery and Medicine, Bern University Hospital, University of Bern, Bern, Switzerland; 6 Department of Surgery, University of Port Harcourt Teaching Hospital, Port Harcourt, Nigeria; 7 Colorectal Surgery Unit, General Surgery Department, Mansoura University Hospitals, Mansoura, Egypt; 8 Department of General Surgery, Hadassah Medical Organization and Faculty of Medicine, Hebrew University of Jerusalem, Jerusalem, Israel; 9 Department of Surgery and Transplantations, Sheba Medical Center, Ramat Gan, Israel

**Keywords:** Trans‑anal minimally invasive surgery (TAMIS), Rigid platforms, Local excision, Early rectal cancer, Systematic review, Meta-analysis

## Abstract

**Background:**

Available platforms for local excision (LE) of early rectal cancer are rigid or flexible [trans‑anal minimally invasive surgery (TAMIS)]. We systematically searched the literature to compare outcomes between platforms.

**Methods:**

PRISMA-compliant search of PubMed and Scopus databases until September 2022 was undertaken in this random-effect meta-analysis. Statistical heterogeneity was assessed using I^2^ statistic. Studies comparing TAMIS versus rigid platforms for LE for early rectal cancer were included. Main outcome measures were intraoperative and short-term postoperative outcomes and specimen quality.

**Results:**

7 studies were published between 2015 and 2022, including 931 patients (423 females); 402 underwent TAMIS and 529 underwent LE with rigid platforms. Techniques were similar for operative time (WMD 11.1, 95%CI − 2.6 to 25, p = 0.11), percentage of defect closure (OR 0.7, 95%CI 0.06–8.22, p = 0.78), and peritoneal violation (OR 0.41, 95%CI 0.12–1.43, p = 0.16). Rigid platforms had higher rates of short-term complications (19.1% vs 14.2, OR 1.6, 95%CI 1.07–2.4, p = 0.02), although no significant differences were seen for major complications (OR 1.41, 95%CI 0.61–3.23, p = 0.41). Patients in the rigid platforms group were 3-times more likely to be re-admitted within 30 days compared to the TAMIS group (OR 3.1, 95%CI 1.07–9.4, p = 0.03). Rates of positive resection margins (rigid platforms: 7.6% vs TAMIS: 9.34%, OR 0.81, 95%CI 0.42–1.55, p = 0.53) and specimen fragmentation (rigid platforms: 3.3% vs TAMIS: 4.4%, OR 0.74, 95%CI 0.33–1.64, p = 0.46) were similar between the groups. Salvage surgery was required in 5.5% of rigid platform patients and 6.2% of TAMIS patients (OR 0.8, 95%CI 0.4–1.8, p = 0.7).

**Conclusion:**

TAMIS or rigid platforms for LE seem to have similar operative outcomes and specimen quality. The TAMIS group demonstrated lower readmission and overall complication rates but did not significantly differ for major complications. The choice of platform should be based on availability, cost, and surgeon’s preference.

**Supplementary Information:**

The online version contains supplementary material available at 10.1007/s00464-024-11065-6.

According to National Comprehensive Network Cancer and European Society of Medical Oncology guidelines, local excision (LE) may be used for treatment of early rectal cancer, defined as T1N0M0 cancer, when specific requirements are satisfied [1, 2]. The modalities used for LE include the traditional Parks' transanal excision for very distal lesions [3], transanal endoscopic microsurgery (TEM) [4] and transanal minimally invasive surgery (TAMIS) [5].

TEM was developed in the 1980s in Germany and involves a rigid platform (rectoscope) through which the lesion is resected using insufflation and custom designed instruments. Similar to TEM is the transanal endoscopic operation (TEO) [5], which was developed around the same time, again involving a rigid platform for resection of rectal lesions. The major difference between the two platforms is that TEO equipment is less costly than that of TEM [6, 7]. TAMIS involves a flexible, disposable platform [4] along with insufflation and standard laparoscopic instruments. We, therefore, aimed to perform a systematic review of the literature to compare the rigid and flexible (TAMIS) platforms in terms of intraoperative outcomes, postoperative morbidity, quality of the specimen and oncologic outcomes.

## Material and methods

### Review registration

This study has been registered in the PROSPERO register of systematic reviews (CRD42022357032) and was reported consistent with the PRISMA 2020 guideline [8]. Ethics approval and written consent to participate in the study were not required given that the study did not include patient information.

### Search strategy and databases searched

**Fig. 1:**
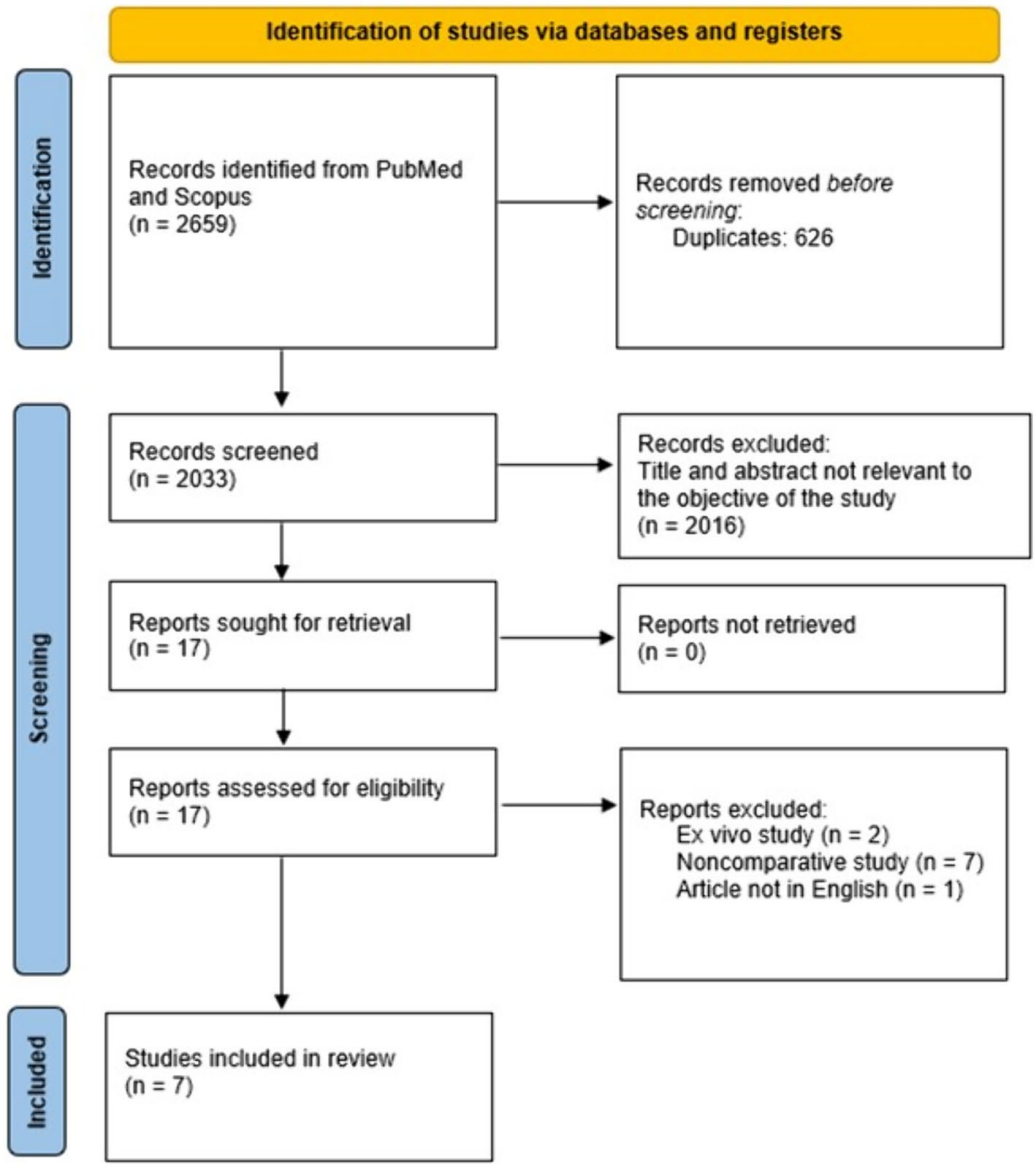
Prisma flowchart

A systematic search of PubMed and Scopus databases was performed through September 2022, by two authors (SMek, SMav). The terms “transanal minimally invasive surgery”, “TEM”, “TEO”, “TES”, “transanal endoscopic”, “microsurgery”, “operation”, “platform”, OR “tool”, “port”, “technique”, “equipment”, "instrument” combined with the Boolean operators AND/OR in order to detect all available studies comparing the two platforms. Following removal of duplicate studies, the abstract list generated by the above search was independently screened by three authors (ZG, SMek, SMav) for potentially relevant studies. After excluding irrelevant papers, a full-text evaluation of all remaining studies was undertaken for completeness and eligibility of reported data, according to the above exclusion criteria (Fig. 1). Any ensuing disagreements were resolved by a third reviewer (SDW).

### Selection criteria

Studies deemed eligible for inclusion had to fulfill the following PICO criteria:

- P (patients): Adult patients undergoing transanal excision of suspicious rectal lesion
- I (intervention): Transanal excision using TAMIS platform
- C (comparator): Transanal excision using rigid platforms (TEM/TEO)
- O (outcome): Specimen quality, operative time, complication rate, recurrence rate, and length of stay.

We excluded studies that included patients younger than 18 years of age, reviews, editorials, clinical vignettes, case reports, animal studies, non-English text, and those with unavailable full-text or that had less than 10 patients.

### Assessment of study quality and risk of bias

The risk of bias across the studies was assessed using the ROBINS-1 tool [9] by two independent authors (PA & ERO). Any conflict of interpretation between the two authors was resolved by a third author (ZG).

### Outcomes

The primary outcome of this review was the specimen quality (fragmentation, R1 resection). Secondary outcomes included postoperative short-term (< 30 days) complications, blood loss, peritoneal violation, rate of defect closure, 30-day readmission, operative time, and oncological outcomes.

### Data collection and analysis

Data of interest included year and quality of the publication, number of patients, sex, maximum diameter of the rectal lesion, complications, time of follow-up, and length of stay, among others. After a thorough full-text evaluation of the included studies, data of interest was extracted into excel spreadsheets (Microsoft, Redmond, Washington, USA) and subsequently cross-checked by two authors (SMek, SMav) for discrepancies.

An open-source, cross-platform software for advanced meta-analysis “openMeta [Analyst] ™” version 12.11.14 was used to conduct the meta-analysis of data. A meta-analysis was conducted to assess the odds ratio (OR) of specimen fragmentation, recurrence, and complications across the studies. Statistical heterogeneity was assessed using the p-value of the Cochrane Q test and the Inconsistency (I^2^) statistics (low if I^2^ < 25%, moderate if I^2^ = 25–75%, and high if I^2^ > 75%).

### Definitions

*Flexible platform:* TAMIS. Includes use of laparoscopic instruments working through an elastic multiport device that is inserted into the anus.

*Rigid platforms for transanal excision:* TEM and TEO. Both use a rigid proctoscope and specialized equipment for the excision.

*Short-term complications:* All complications recorded ≤ 30 days postoperatively.

*Minor Complications:* Complications not requiring intervention under general anesthesia (Clavien-Dindo < IIIb according) [10].

*Major Complications:* Complications requiring intervention under general anesthesia (Clavien-Dindo ≥ IIIb) [10].

*Salvage Surgery:* Additional surgery to address residual or recurrent disease.

## Results

### Study and patient characteristics

A total of 7 [11–17] studies published between 2015 and 2022 were included in the analysis, which encompassed 931 patients [423 females; median age 63 (range 20–92) years]. 402 patients underwent TAMIS, while 529 underwent transanal excision with the use of rigid platforms (TEM and TEO). The median age and body mass index (BMI) were comparable between the two groups (Table 1). Three studies [12, 13, 15] report details on neoadjuvant radiation therapy that was given to 36 (6.8%) patients in the rigid platforms group and 23 patients (5.7%) in the TAMIS group. Only one study [17] involved 10 patients who underwent robotic TAMIS. The outcomes of the robotic TAMIS platform were aggregated with conventional TAMIS and compared to the rigid platforms.

**Table 1:** Studies and patient characteristics

Studies	Year	Total No patients	TEM/TEO		TAMIS	
			No females	Age (mean)	No females	Age (mean)
Molina et al. [14]	2015	78	42	61.4	N/A	61.4
Melin et al. [11]	2016	69	18	63.2	12	64.3
Lee et al. [13]	2017	428	97	65.9	74	65.0
Mege et al. [12]	2017	74	13	63	15	67
Van den Eynde et al. [16]	2019	121	41	63	45	69
Stipa et al. [15]	2022	132	30	66.8	29	67.1
Schwab et al. [17]	2022	29	7	56	9	NA

*TEM/TEO* transanal endoscopic microsurgery/transanal endoscopic operation, *TAMIS* trans‑anal minimally invasive surgery

### Intraoperative outcomes

**Fig. 2:**
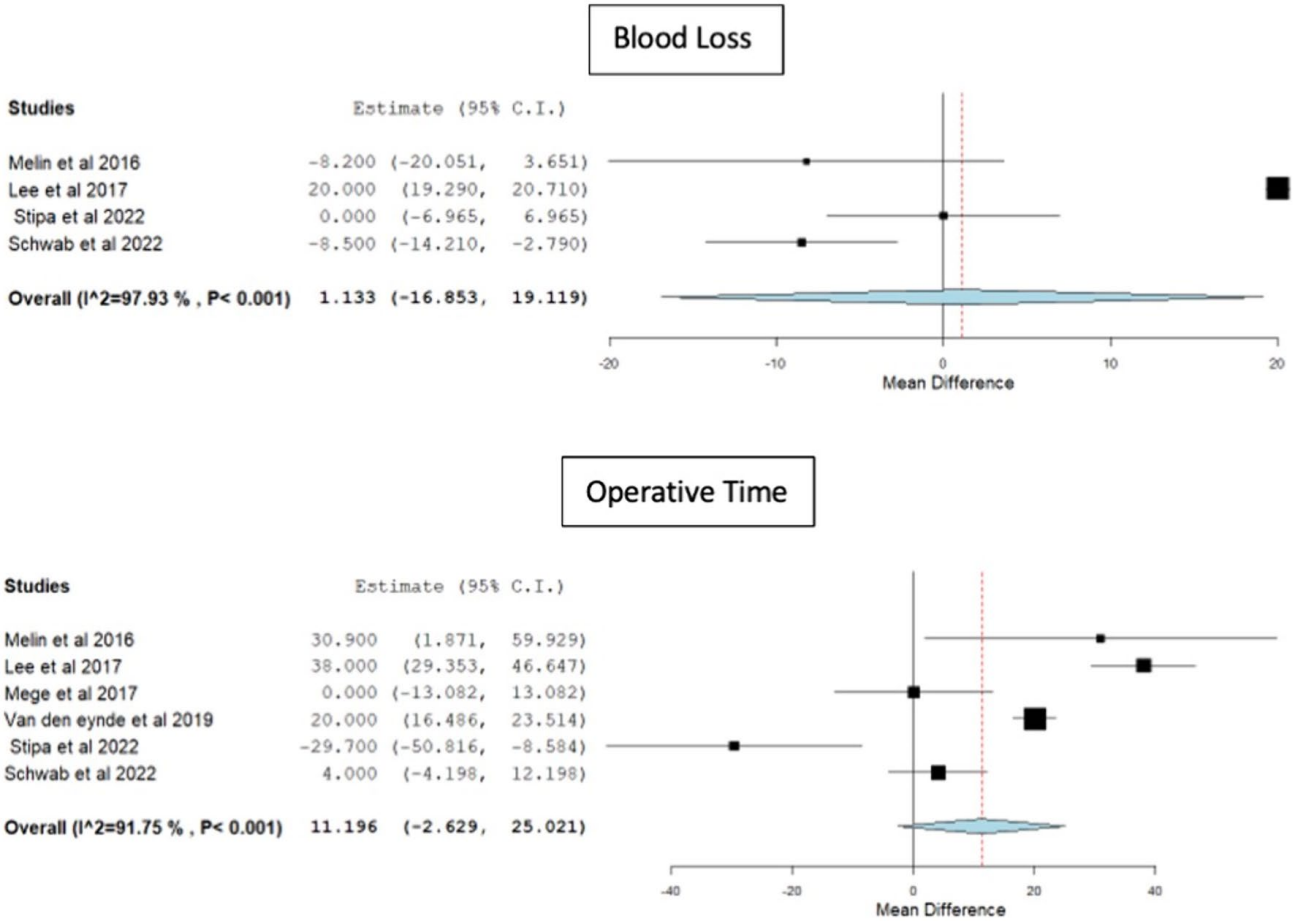
Forest plots for blood loss and operative time

**Fig. 3:**
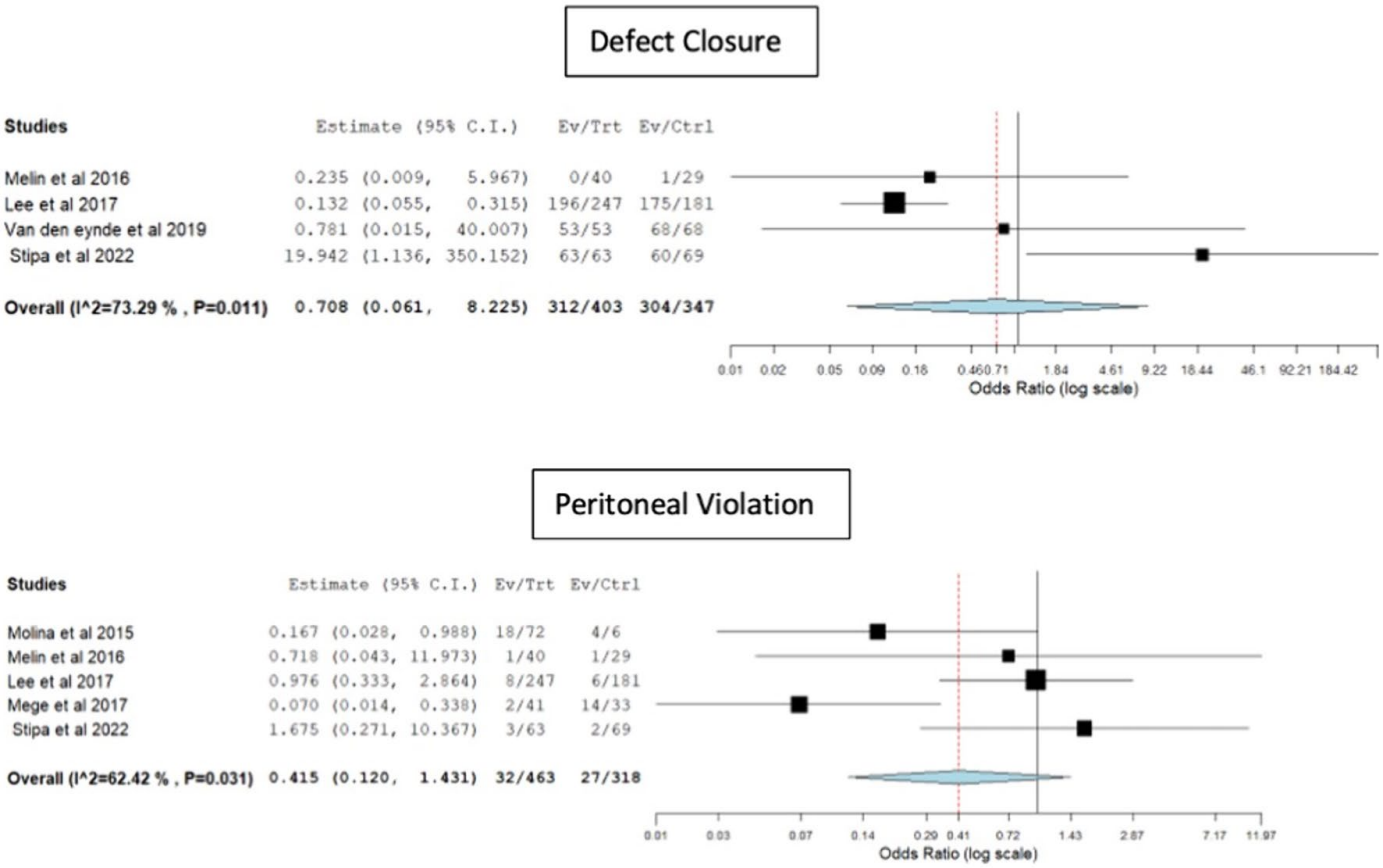
Forest plots for defect closure and peritoneal violation

There were no significant differences observed between TAMIS and rigid platforms in terms of blood loss [weighted mean difference (WMD) 1.13, 95% CI − 16.8 to 19.1, p = 0.9, I^2^ = 97.9] or operative time (WMD 11.1, 95% CI − 2.6 to 25, p = 0.11, I^2^ = 91.7) (Fig. 2). The odds of defect closure (OR 0.7, 95% CI 0.06–8.22, p = 0.78, I^2^ = 73.2) and peritoneal violation (OR 0.41, 95% CI 0.12–1.43, p = 0.16, I^2^ = 62.4) were similar between the two groups (Fig. 3). Only 4 studies [11, 13–15] provided information on conversion to an abdominal approach after a peritoneal violation. Eight (18.6%) out of 43 patients were converted to an abdominal approach after the peritoneal violation [5/30 (16.7%) in the rigid platforms group and 3/13 (23.1%) in the TAMIS group].

### Short-term post-operative outcomes

**Fig. 4:**
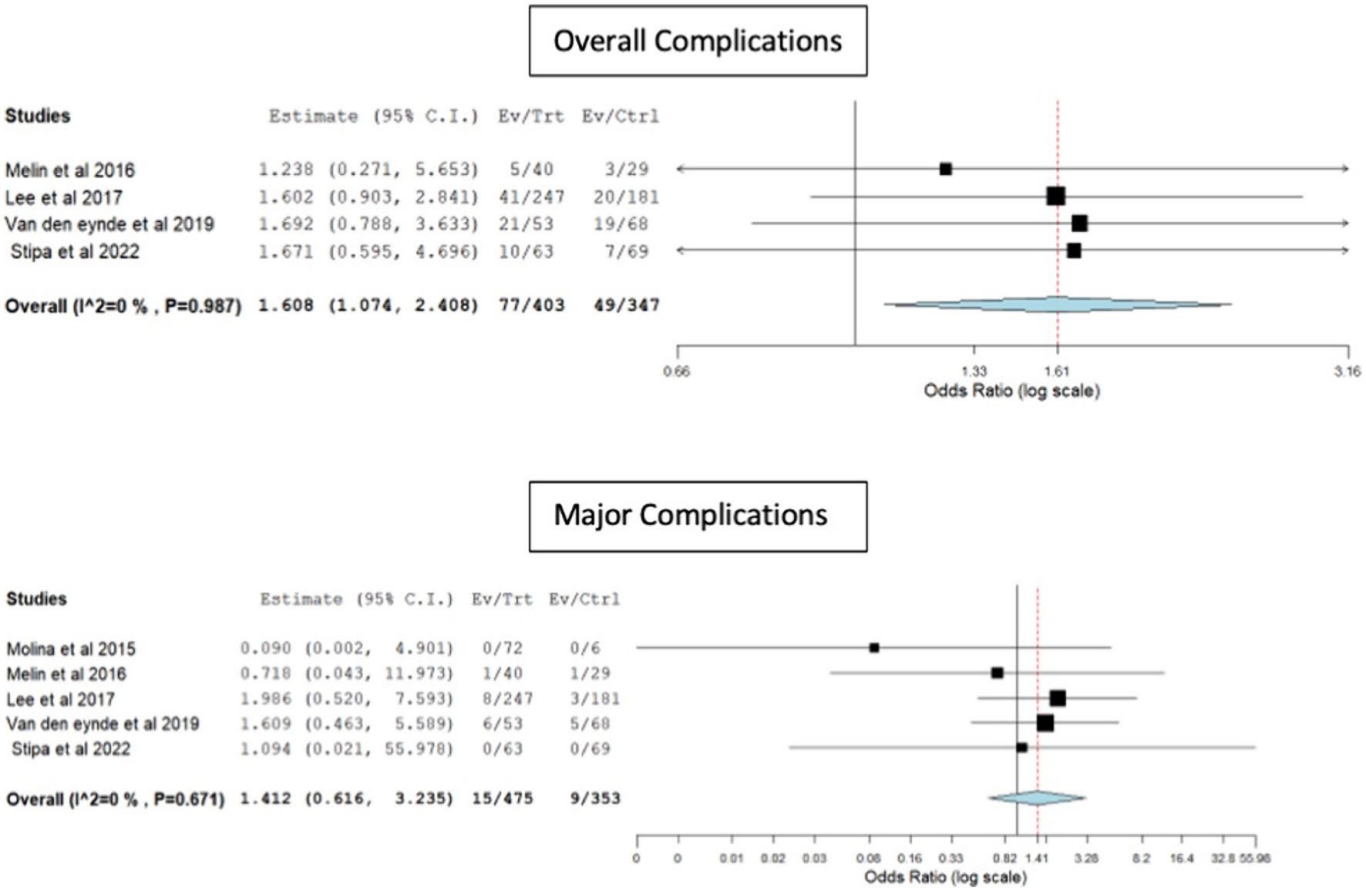
Forest plots for overall and major complications

Rigid platforms had a higher rate of short-term complications (19.1% vs 14.2, OR 1.6, 95% CI 1.07–2.4, p = 0.02, I^2^ = 0). There were no significant differences between the two techniques in terms of minor (OR 1.52, 95% CI 0.99–2.36, p = 0.056, I^2^ = 0) or major (OR 1.41, 95% CI 0.61–3.23, p = 0.41, I^2^ = 0) complications (Fig. 4).

**Fig. 5:**
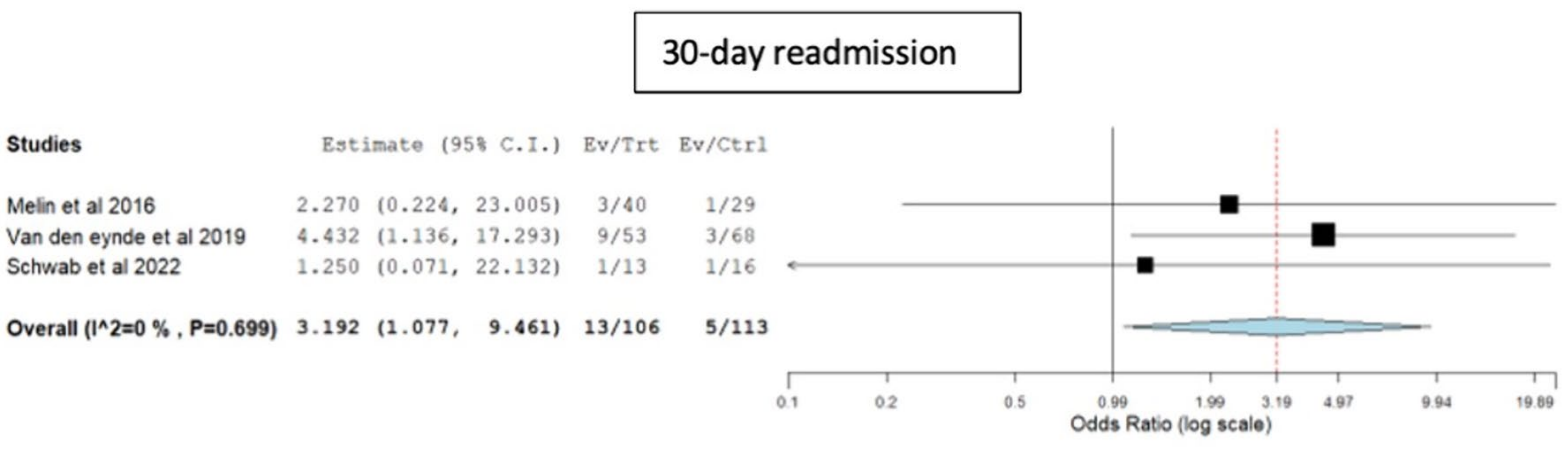
Forest plot for 30-day readmission rate

The re-admission rate within 30 days was higher in the rigid platforms group, with patients being 3-times more likely to be re-admitted compared to the TAMIS group (OR 3.1, 95% CI 1.07–9.4, p = 0.03, I^2^ = 0) (Fig. 5).

### Pathologic and oncological outcomes

**Fig. 6:**
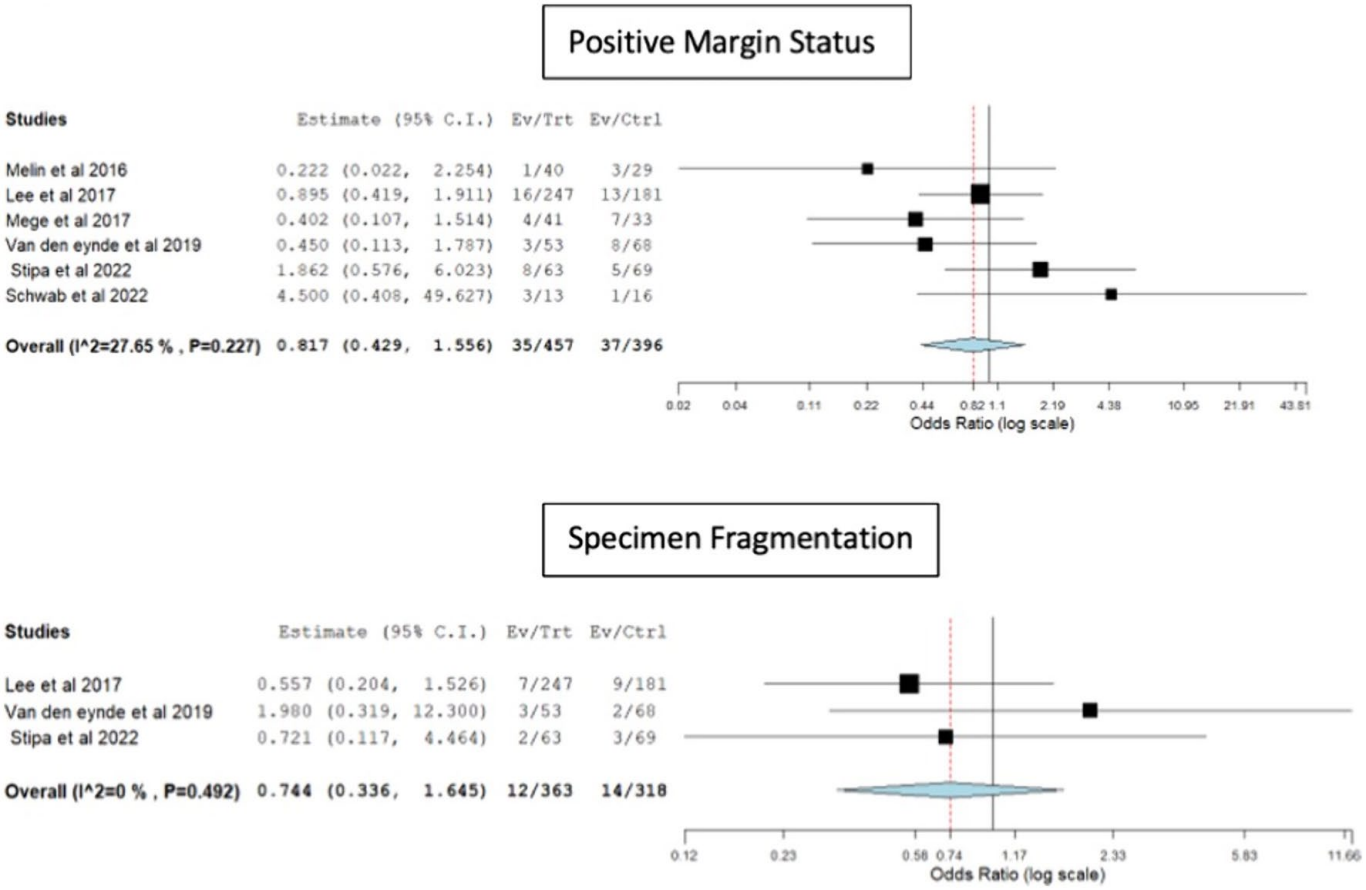
Forest plots for positive resection margins and specimen fragmentation

The rates of positive resection margins (rigid platforms: 7.6% vs TAMIS: 9.34%, OR 0.81, 95% CI 0.42–1.55, p = 0.53, I^2^ = 27.6) and specimen fragmentation (rigid platforms: 3.3% vs TAMIS: 4.4%, OR 0.74, 95% CI 0.33–1.64, p = 0.46, I^2^ = 0) were similar in both groups (Fig. 6).

Analysis of oncological outcomes from three studies with a median follow-up of 14 months showed comparable rates of local recurrence (rigid platforms: 3.2% vs TAMIS: 3.9%, OR 0.8, 95% CI 0.33–1.94, p = 0.63, I^2^ = 0). The rates of salvage surgery were 5.5% for rigid platforms and 6.2% for TAMIS (OR 0.8, 95% CI 0.4–1.8, p = 0.7, I^2^ = 0).

### Quality of the studies and certainty of evidence

All studies were deemed as serious risk of bias (Supplementary Table 1) according to ROBINS-1 tool [9] and therefore the quality of evidence provided for all outcomes in this meta-analysis was assessed as very low according to the GRADE approach (Supplementary Table 2) [18]

## Discussion

Early rectal cancer with favorable histology is amenable to treatment with LE if specific criteria are met [1, 2]. Local excision for rectal cancer has traditionally been performed transanally. Historically, the initial approach to LE was the Parks’ transanal excision. However, this option evolved to encompass both rigid (TEM and TEO) and flexible platforms (TAMIS). A meta-analysis of comparative non-randomized trials published in 2015 by Clancy et al. [19] demonstrated that TEM was associated with better outcomes in terms of specimen quality, which translated into a reduced rate of specimen fragmentation and a lower rate of positive microscopic margins (R1) when compared to classic transanal excision. Moreover, the authors noted that recurrence rates were lower in the TEM group [19].

The first platforms used for endoscopic transanal excision, developed in Germany during the 1980s, were rigid in their design (TEM) [4]. Subsequently, a similar rigid platform was produced by Karl Storz and was introduced as TEO [20]. Both of these platforms shared similar properties including rigid resectoscopes with insufflation mechanisms and custom designed instruments. There are three major disadvantages with these platforms: [7] high cost as they require specialized equipment (insufflator, resectoscope, stereotactic scope, and angled instruments); length of the platform (longer than the flexible one by 10–15 cm) allows less triangulation of the working instruments; and the view is static given that the scope is fixed in a specific position. The latter two characteristics are what make the TEM and TEO procedures more technically demanding [7]. In 2010 Atallah et al. [5] presented a novel transanal approach using the single-incision laparoscopic surgery port (SILS Port, Covidien). A tailor-made platform (GelPoint, Path Transanal Access Platform) was subsequently marketed to allow the use of standard laparoscopic instruments and insufflators.

The main aim of our study was to compare the intraoperative outcomes of the rigid and flexible platforms for transanal excision. We did not find any significant differences between the two platforms in terms of blood loss or operative time. The learning curve for TAMIS is a minimum of 14–24 cases to reach reported R0 resection rates according to Lee et al. [21], while the learning curve for TEM ranges in the literature from 4 [22] to more than 36 cases [23]. Interestingly, although TEM and TEO are considered more technically demanding and associated with a steeper learning curve, our study did not find any differences in defect closure, peritoneal violation, or specimen fragmentation. Although the median follow-up of our study was too short to make any definitive conclusions regarding oncologic outcomes, local recurrence rates were similar between the two platforms. Similarly, R1 resection rates and rates of salvage surgery were similar between the two platforms.

Common postoperative complications of both platforms reported in the literature are acute urinary retention (4.9%) and rectal bleeding (2.2%) [24]. According to the results of our study, the TAMIS group demonstrated lower rates of overall complications. Nevertheless, the two platforms did not significantly differ in terms of major complications (rigid platforms group: 3.1% versus TAMIS: 2.54%). The rigid platform group demonstrated a higher rate of minor complications (12.65%) compared to TAMIS (10%), although this difference did not reach statistical significance (OR 1.52, 95% CI 0.99–2.36, p = 0.056). This might explain the higher 30-day re-admission rate in the rigid platforms group (OR 3.1, 95% CI 1.07–9.4, p = 0.03).

Unfortunately, the studies included in this review did not report any data regarding cost or functional results. A systematic review by Marinello et al. [25] in 2020 showed some short-term deterioration in manometric results using either platform but with no impairment in quality of life. Nevertheless, the authors state that these results should be interpreted with caution as the data were heterogeneous and pooled analysis was not feasible. Another case series [26] published later the same year, including only patients undergoing TAMIS with a 5-year follow-up, showed that the majority of the patients (approximately 73%) had no symptoms of low anterior resection syndrome after five years. Regarding cost-effectiveness, Yu et al. [27] using a Markov model, reported similar cost-effectiveness among TEM and TAMIS, even though the initial capital for obtaining TEM and TEO equipment is much higher than TAMIS.

The two platform types demonstrate comparable intraoperative outcomes and specimen quality. TAMIS seems to be associated with a lower readmission rate and overall complication rate. Nevertheless, our study has certain limitations. All studies were retrospective, thus amenable to selection bias and involving a relatively low number of patients. In addition, the surgeon’s experience with each platform is not reported, which could be a significant confounding factor. Finally, although these procedures are better used for patients with early rectal cancer, they have also been employed for palliative reasons and for patients with more advanced disease who were not deemed eligible to undergo surgery. Thus, we did not focus on long-term oncologic outcomes.

## Conclusion

The use of either TAMIS or rigid platforms for LE of early rectal cancer seem to have similar operative and short-term postoperative outcomes, except for a lower readmission rate and lower overall complication rate after TAMIS. Moreover, specimen quality did not differ between the two techniques. The certainty of evidence was very low, which unfortunately precluded our ability to recommend any one technique. Thus, the choice of platform should be based on its availability, costs, and surgeon’s preference and experience.

### Supplementary Information

Below is the link to the electronic supplementary material.

Supplementary file1 (DOCX 18 kb)

Supplementary file2 (DOCX 20 kb)

## References

[1] https://www.nccn.org/professionals/physician_gls/pdf/rectal.pdf. Accessed 26 June 2023

[2] Glynne-Jones R, Wyrwicz L, Tiret E (2017) Rectal cancer: ESMO Clinical Practice Guidelines for diagnosis treatment and follow-up. Ann Oncol 28:iv22–iv40 28881920 10.1093/annonc/mdx224

[3] Parks AG, Stuart AE (1973) The management of villous tumours of the large bowel. Br J Surg 60:688–695. 10.1002/bjs.1800600908 4582241 10.1002/bjs.1800600908

[4] Buess G, Theiss R, Hutterer F et al (1983) Transanal endoscopic surgery of the rectum—testing a new method in animal experiments. Leber Magen Darm 13:73–77 6621245

[5] Atallah S, Albert M, Larach S (2010) Transanal minimally invasive surgery: a giant leap forward. Surg Endosc 24:2200–2205 20174935 10.1007/s00464-010-0927-z

[6] D’Hondt M, Yoshihara E, Dedrye L, Vindevoghel K, Nuytens F, Pottel H (2017) Transanal endoscopic operation for benign rectal lesions and T1 carcinoma. JSLS 21(e2016):00093. 10.4293/JSLS.2016.00093 10.4293/JSLS.2016.00093 PMC5266515 28144126

[7] Devane LA, Daly MC, Albert MR (2022) Transanal endoscopic platforms: TAMIS versus rigid platforms: pros and cons. Clin Colon Rectal Surg 35:93–98. 10.1055/s-0041-1742108 35237103 10.1055/s-0041-1742108 PMC8885160

[8] Page MJ, McKenzie JE, Bossuyt PM, et al (2021) The PRISMA 2020 statement: an updated guideline for reporting systematic reviews. BMJ 372:n71. 10.1136/bmj.n71 33782057 10.1136/bmj.n71 PMC8005924

[9] Sterne JA, Hernan MA, Reeves BC, et al (2016) ROBINS-I: a tool for assessing risk of bias in non-randomised studies of interventions. BMJ 355:i4919. 10.1136/bmj.i4919 27733354 10.1136/bmj.i4919 PMC5062054

[10] Clavien PA, Barkun J, de Oliveira ML et al (2009) The Clavien-Dindo classification of surgical complications: five-year experience. Ann Surg 250:187–196. 10.1097/SLA.0b013e3181b13ca2 19638912 10.1097/SLA.0b013e3181b13ca2

[11] Melin AA, Kalaskar S, Taylor L, Thompson JS, Ternent C, Langenfeld SJ (2016) Transanal endoscopic microsurgery and transanal minimally invasive surgery: is one technique superior? Am J Surg 212:1063–1067. 10.1016/j.amjsurg.2016.08.017 27810138 10.1016/j.amjsurg.2016.08.017

[12] Mege D, Bridoux V, Maggiori L, Tuech JJ, Panis Y (2017) What is the best tool for transanal endoscopic microsurgery (TEM)? A case-matched study in 74 patients comparing a standard platform and a disposable material. Int J Colorectal Dis 32:1041–1045. 10.1007/s00384-016-2733-0 28011978 10.1007/s00384-016-2733-0

[13] Lee L, Edwards K, Hunter IA et al (2017) Quality of local excision for rectal neoplasms using transanal endoscopic microsurgery versus transanal minimally invasive surgery: a multi-institutional matched analysis. Dis Colon Rectum 60:928–935. 10.1097/DCR.0000000000000884 28796731 10.1097/DCR.0000000000000884

[14] Molina G, Bordeianou L, Shellito P, Sylla P (2016) Transanal endoscopic resection with peritoneal entry: a word of caution. Surg Endosc 30:1816–1825. 10.1007/s00464-015-4452-y 26264697 10.1007/s00464-015-4452-y

[15] Stipa F, Tierno SM, Russo G, Burza A (2022) Trans-anal minimally invasive surgery (TAMIS) versus trans-anal endoscopic microsurgery (TEM): a comparative case-control matched-pairs analysis. Surg Endosc 36:2081–2086. 10.1007/s00464-021-08494-y 33844090 10.1007/s00464-021-08494-y

[16] Van den Eynde F, Jaekers J, Fieuws S, D’Hoore AM, Wolthuis AM (2019) TAMIS is a valuable alternative to TEM for resection of intraluminal rectal tumors. Tech Coloproctol 23:161–166. 10.1007/s10151-019-01954-7 30859349 10.1007/s10151-019-01954-7

[17] Schwab ME, Hernandez S, Watanaskul S, Chern H, Varma M, Sarin A (2022) Comparison of advanced techniques for local excision of rectal lesions: a case series. BMC Surg 22:117. 10.1186/s12893-022-01543-w 35346146 10.1186/s12893-022-01543-w PMC8962117

[18] Schünemann H, Brożek J, Guyatt G, Oxman A, eds (2013) GRADE handbook for grading quality of evidence and strength of recommendations. Updated October 2013. The GRADE Working Group

[19] Clancy C, Burke JP, Albert MR, O’Connell PR, Winter DC (2015) Transanal endoscopic microsurgery versus standard transanal excision for the removal of rectal neoplasms: a systematic review and meta-analysis. Dis Colon Rectum 58:254–261. 10.1097/DCR.0000000000000309 25585086 10.1097/DCR.0000000000000309

[20] Hur H, Bae SU, Han YD et al (2016) Transanal endoscopic operation for rectal tumor: short-term outcomes and learning curve analysis. Surg Laparosc Endosc Percutan Tech 26:236–243. 10.1097/SLE.0000000000000258 27077220 10.1097/SLE.0000000000000258

[21] Lee L, Kelly J, Nassif GJ et al (2018) Establishing the learning curve of transanal minimally invasive surgery for local excision of rectal neoplasms. Surg Endosc 32:1368–1376. 10.1007/s00464-017-5817-1 28812153 10.1007/s00464-017-5817-1

[22] Maya A, Vorenberg A, Oviedo M, da Silva G, Wexner SD, Sands D (2014) Learning curve for transanal endoscopic microsurgery: a single-center experience. Surg Endosc 28:1407–1412. 10.1007/s00464-013-3341-5 24366188 10.1007/s00464-013-3341-5

[23] Barendse RM, Dijkgraaf MG, Rolf UR et al (2013) Colorectal surgeons’ learning curve of transanal endoscopic microsurgery. Surg Endosc 27:3591–3602. 10.1007/s00464-013-2931-6 23572216 10.1007/s00464-013-2931-6

[24] Serra-Aracil X, Badia-Closa J, Pallisera-Lloveras A et al (2021) Management of intra- and postoperative complications during TEM/TAMIS procedures: a systematic review. Minerva Surg 76:343–349. 10.23736/S2724-5691.20.08405-9 33433070 10.23736/S2724-5691.20.08405-9

[25] Marinello FG, Curell A, Tapiolas I, Pellino G, Vallribera F, Espin E (2020) Systematic review of functional outcomes and quality of life after transanal endoscopic microsurgery and transanal minimally invasive surgery: a word of caution. Int J Colorectal Dis 35:51–67. 10.1007/s00384-019-03439-3 31761962 10.1007/s00384-019-03439-3

[26] Goldenshluger M, Gutman Y, Katz A (2020) Long-term bowel function after transanal minimally invasive surgery (TAMIS). Isr Med Assoc J 22:426–430 33236567

[27] Yu JX, Russell WA, Ching JH, Kim N, Bendavid E, Owens DK, Kaltenbach T (2019) Cost effectiveness of endoscopic resection vs transanal resection of complex benign rectal polyps. Clin Gastroenterol Hepatol 17(13):2740–2748.e6. 10.1016/j.cgh.2019.02.041 30849517 10.1016/j.cgh.2019.02.041

