# Supplementary material for: Trans‑anal minimally invasive surgery (TAMIS) versus rigid platforms for local excision of early rectal cancer: a systematic review and meta-analysis of the literature
Source: Surg Endosc. 2024 Jul 18;38(8):4198–206. doi: 10.1007/s00464-024-11065-6 (PMC11289048; doi:10.1007/s00464-024-11065-6)
Supplement: Supplementary file 1 — Supplementary file1 (DOCX 18 kb) [file 464_2024_11065_MOESM1_ESM.docx]

| **Included studies** | **Bias due to confounding** | **Bias in selection of participants in the study** | **Bias in classification of intervention** | **Bias due to deviation from the intended intervention** | **Bias due to missing data** | **Bias in measurement of outcomes** | **Bias in selection of the reported result** | **Overall risk** |
| --- | --- | --- | --- | --- | --- | --- | --- | --- |
| **Molina et al 2015** | Serious | Serious | Low | Low | Low | Low | Low | Serious |
| **Melin et al 2016** | Serious | Serious | Low | Low | Low | Low | Low | Serious |
| **Lee et al 2017** | Serious | Serious | Low | Low | Low | Low | Low | Serious |
| **Lee et al. 2017** | Serious | Serious | Low | Low | Moderate | Low | Low | Serious |
| **Van den Eynde et al. 2019** | Serious | Serious | Low | Low | Low | Low | Low | Serious |
| **Stipa et al. 2022** | Serious | Serious | Low | Low | Low | Low | Serious | Serious |
| **Schwab et al. 2022** | Serious | Serious | Low | Low | Moderate | Moderate | Low | Serious |

**Appendix Table (Results of quality assessment of non-randomized studies using ROBINS-1 tool)**
