# Supplementary material for: Trans‑anal minimally invasive surgery (TAMIS) versus rigid platforms for local excision of early rectal cancer: a systematic review and meta-analysis of the literature
Source: Surg Endosc. 2024 Jul 18;38(8):4198–206. doi: 10.1007/s00464-024-11065-6 (PMC11289048; doi:10.1007/s00464-024-11065-6)
Supplement: Supplementary file 2 — Supplementary file2 (DOCX 20 kb) [file 464_2024_11065_MOESM2_ESM.docx]

| **Certainty assessment** | | | | | | | **№ of patients** | | **Effect** | | **Certainty** |  |
| --- | --- | --- | --- | --- | --- | --- | --- | --- | --- | --- | --- | --- |
| **№ of studies** | **Study design** | **Risk of bias** | **Inconsistency** | **Indirectness** | **Imprecision** | **Other considerations** | **TAMIS** | **rigid platforms** | **Relative (95% CI)** | **Absolute (95% CI)** |  |  |
| **Peritoneal Violation** | | | | | | | | | | | | |
| 5 | non-randomised studies | very serious^a^ | very serious^b^ | not serious | not serious | none | 27/318 (8.5%) | 32/463 (6.9%) | **OR 0.40** (0.12 to 1.40) | **40 fewer per 1,000** (from 60 fewer to 25 more) | ⨁◯◯◯ Very low |  |
| **Defect Closure** | | | | | | | | | | | | |
| 4 | non-randomised studies | extremely serious^a^ | very serious^c^ | not serious | not serious | none | 304/347 (87.6%) | 312/403 (77.4%) | **OR 0.70** (0.06 to 8.20) | **68 fewer per 1,000** (from 604 fewer to 191 more) | ⨁◯◯◯ Very low |  |
| **Blood Loss** | | | | | | | | | | | | |
| 4 | non-randomised studies | extremely serious^a^ | very serious^d^ | not serious | not serious | none |  |  | - | **0**  (0 to 0 ) | ⨁◯◯◯ Very low |  |
| **Overall Complications** | | | | | | | | | | | | |
| 4 | non-randomised studies | extremely serious^a^ | not serious | not serious | not serious | none | 49/347 (14.1%) | 77/403 (19.1%) | **OR 1.60** (1.07 to 2.40) | **83 more per 1,000** (from 11 more to 171 more) | ⨁◯◯◯ Very low |  |
| **Minor Complications** | | | | | | | | | | | | |
| 4 | non-randomised studies | extremely serious^a^ | not serious | not serious | not serious | none | 40/347 (11.5%) | 62/403 (15.4%) | **OR 1.5** (0.9 to 2.3) | **60 more per 1,000** (from 13 fewer to 141 more) | ⨁◯◯◯ Very low |  |
| **Major Complications** | | | | | | | | | | | | |
| 5 | non-randomised studies | extremely serious^a^ | not serious | not serious | not serious | none | 9/353 (2.5%) | 15/475 (3.2%) | **OR 1.4** (0.6 to 3.2) | **12 more per 1,000** (from 12 fewer to 63 more) | ⨁◯◯◯ Very low |  |
| **30-day readmission** | | | | | | | | | | | | |
| 3 | non-randomised studies | extremely serious^a^ | not serious | not serious | not serious | none | 5/113 (4.4%) | 13/106 (12.3%) | **OR 3.19** (1.00 to 9.40) | **186 more per 1,000** (from 0 fewer to 445 more) | ⨁◯◯◯ Very low |  |
| **Lesion Fragmentation** | | | | | | | | | | | | |
| 3 | non-randomised studies | extremely serious^a^ | not serious | not serious | not serious | none | 14/318 (4.4%) | 12/363 (3.3%) | **OR 0.7** (0.3 to 1.6) | **10 fewer per 1,000** (from 23 fewer to 19 more) | ⨁◯◯◯ Very low |  |
| **Salvage Surgery** | | | | | | | | | | | | |
| 3 | non-randomised studies | extremely serious^a^ | not serious | not serious | not serious | none | 15/243 (6.2%) | 18/328 (5.5%) | **OR 0.80** (0.42 to 1.80) | **10 fewer per 1,000** (from 31 fewer to 40 more) | ⨁◯◯◯ Very low |  |
| **Local Recurrence** | | | | | | | | | | | | |
| 3 | non-randomised studies | extremely serious^a^ | not serious | not serious | not serious | none | 11/278 (4.0%) | 11/340 (3.2%) | **OR 0.8** (0.3 to 1.9) | **6 fewer per 1,000** (from 22 fewer to 27 more) | ⨁◯◯◯ Very low |  |
| **Positive Margins** | | | | | | | | | | | | |
| 6 | non-randomised studies | extremely serious^a^ | not serious | not serious | not serious | none | 37/396 (9.3%) | 35/457 (7.7%) | **OR 0.81** (0.40 to 1.50) | **14 fewer per 1,000** (from 44 fewer to 34 more) | ⨁◯◯◯ Very low |  |

**CI:** confidence interval; **OR:** odds ratio

#### Explanations

a. The studies were assessed as serious risk according to ROBINS-I

b. I2=62.4%

c. I2=73.29%

d. I2=97.9%
